# Differential Regulation of GPCRs—Are GRK Expression Levels the Key?

**DOI:** 10.3389/fcell.2021.687489

**Published:** 2021-05-24

**Authors:** Edda S. F. Matthees, Raphael S. Haider, Carsten Hoffmann, Julia Drube

**Affiliations:** Institut für Molekulare Zellbiologie, CMB – Center for Molecular Biomedicine, Universitätsklinikum Jena, Friedrich-Schiller-Universität Jena, Jena, Germany

**Keywords:** GPCR, GRK, β-arrestin, IDP, tissue-specific expression, barcode hypothesis

## Abstract

G protein-coupled receptors (GPCRs) comprise the largest family of transmembrane receptors and their signal transduction is tightly regulated by GPCR kinases (GRKs) and β-arrestins. In this review, we discuss novel aspects of the regulatory GRK/β-arrestin system. Therefore, we briefly revise the origin of the “barcode” hypothesis for GPCR/β-arrestin interactions, which states that β-arrestins recognize different receptor phosphorylation states to induce specific functions. We emphasize two important parameters which may influence resulting GPCR phosphorylation patterns: (A) direct GPCR–GRK interactions and (B) tissue-specific expression and availability of GRKs and β-arrestins. In most studies that focus on the molecular mechanisms of GPCR regulation, these expression profiles are underappreciated. Hence we analyzed expression data for GRKs and β-arrestins in 61 tissues annotated in the Human Protein Atlas. We present our analysis in the context of pathophysiological dysregulation of the GPCR/GRK/β-arrestin system. This tissue-specific point of view might be the key to unraveling the individual impact of different GRK isoforms on GPCR regulation.

## Introduction

**FIGURE 1:**
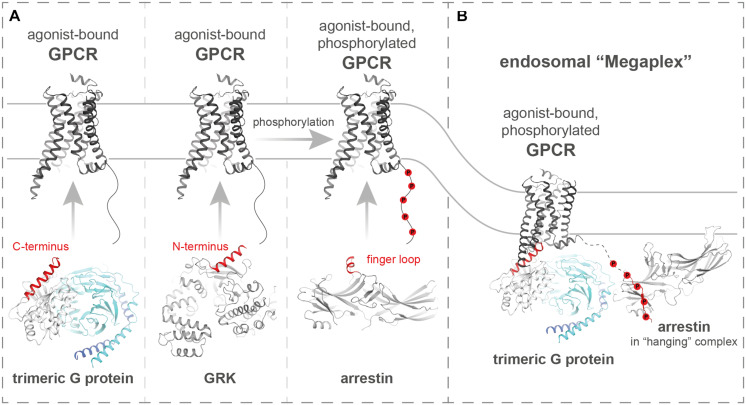
G proteins, GRKs, and arrestins share similar structural features to engage active GPCR folds. **(A)** Schematic depiction of how active GPCRs (PDB: 3SN6) interact with trimeric G proteins (PDB: 3SN6), GRKs (PDB: 3NYN), and arrestins (PDB: 5DGY), as most prominent mediators and regulators of GPCR signaling. On the cartoon structures of the intracellular effector proteins, helices that interact with active GPCR folds are highlighted in red. **(B)** Structure of the endosomal complex (PDB: 6NI2) between an active GPCR, a trimeric G protein, and β-arrestin1.

G protein-coupled receptors (GPCRs) constitute a family of over 800 membrane-localized receptors. They respond to a large variety of extracellular stimuli, among them, photons, odors, hormones, or neurotransmitters, to induce specific intracellular signaling (Marinissen and Gutkind, 2001). This is achieved by a vast diversity of ligand binding domains. Nevertheless, GPCRs share a seven-transmembrane architecture that undergoes large conformational changes during receptor activation in order to activate a common set of intracellular signaling proteins (Nygaard et al., 2013; Latorraca et al., 2017). Hence, G proteins, GPCR kinases (GRKs) and arrestins, as most prominent interaction partners of GPCRs, engage active receptors at their opened intracellular cavity in a similar fashion (Nygaard et al., 2013; Flock et al., 2017). This process usually involves the insertion of small loop structures or alpha-helical domains into the GPCR cavity. The similarities between the C-terminal alpha helix of G_α_ subunits, the N-terminal domain of GRKs, and the finger loop region (FLR) of arrestins, which enable or enhance the interaction with active GPCRs, are highlighted in Figure 1A.

For the main signaling transducers, the trimeric G proteins, this interaction leads to a guanosine diphosphate (GDP)—guanosine triphosphate (GTP) exchange followed by dissociation of G_α_ and G_βγ_ subunits (Oldham and Hamm, 2008; Flock et al., 2015). The now activated G protein subunits are able to individually regulate levels of second messengers [e.g., cyclic adenosine monophosphate (cAMP), calcium, or diacylglycerol (DAG)] to induce a cellular response. Subsequently, intracellular peptide stretches of active GPCRs are phosphorylated by GRKs. In turn, this accumulation of negative charges enables high affinity binding of arrestins (Gurevich and Gurevich, 2019), initiating the desensitization and internalization of receptors. As arrestins and G proteins utilize at least overlapping binding interfaces (DeWire et al., 2007), arrestin-bound receptors are canonically unable to further induce their primary signaling. Moreover, arrestins have been shown to serve as scaffolds for more than 100 intracellular proteins (Xiao et al., 2007; Crepieux et al., 2017), that enable the formation of specific effector-hubs, regulating intracellular trafficking and signaling of active GPCRs. In this review, we want to discuss the current state of research regarding the phosphorylation-dependent processes that underlie GPCR regulation. Moreover, we want to highlight the potential influence of tissue specific expression levels of GPCR-regulating genes on signaling outcomes.

## Arrestins and GRKs Facilitate Targeted Downstream Functions for Hundreds of GPCRs

Human physiology features a sizeable amount of G_α_ and G_βγ_ subunits (Milligan and Kostenis, 2006). Thus, the diversity of primary GPCR signaling is adequately explained as different receptors preferably couple to specific combinations of G protein trimers (Inoue et al., 2019). However, the downregulation of most GPCRs is tightly controlled by only four ubiquitously expressed GRKs (GRK2, 3, 5, and 6) and two arrestin isoforms, namely β-arrestin1 and β-arrestin2. Still, the processes enabled by these proteins are highly diverse and seem specific for each GPCR. For some receptors, the interactions with GRKs and arrestins lead to desensitization and immediate recycling, redirecting the receptor back to the membrane after initial internalization (Claing et al., 2002). In contrast, certain GPCRs exhibit prolonged intracellular trafficking which localizes the receptors to specific intracellular compartments and may give rise to a second wave of endosomal signaling (Godbole et al., 2017).

GRKs have been shown to be allosterically activated via binding to active GPCRs (Palczewski et al., 1991; Chen et al., 1993; Huang and Tesmer, 2011). This binding mechanism has not been fully understood yet, but possibly features the insertion of a N-terminal α-helix into the cytoplasmic cavity of the GPCR. Although structural evidence is not necessarily conclusive (Cato et al., 2021), this mode of GRK-binding is highly attractive, as G proteins and arrestins probe for active GPCR conformations in a similar fashion (Figure 1A). In a cellular context, GRK-binding leads to the phosphorylation of active GPCRs at their intracellular sites. Notably, GRKs have also been shown to phosphorylate non-GPCR substrates (Palczewski et al., 1991; McCarthy and Akhtar, 2002), albeit with higher efficiency in the presence of active GPCRs. Thus, GRKs most likely also regulate other cellular processes in a phosphorylation-dependent manner, but in this review, we will predominantly discuss their impact on GPCR signaling. Non-visual GRKs are classified into two families (Gurevich et al., 2012; Mushegian et al., 2012; Homan and Tesmer, 2014). GRK2 and GRK3 constitute the GRK2 family and are expressed in the cytosol. Subsequent to GPCR activation, GRK2 and 3 are recruited to the membrane, facilitated by GPCR complex formation and stabilizing interactions with G_βγ_-subunits (Tesmer et al., 2005). In contrast, GRK4 family kinases, namely GRK4, 5, and 6, are generally membrane-associated. In this review, we will further focus on effects of GRK2, 3, 5, and 6. Some of these GRK isoforms have been shown to preferentially phosphorylate different residues at the intracellular side of GPCRs (Nobles et al., 2011), to induce receptor internalization and desensitization.

Upon binding to the active and phosphorylated GPCR, arrestins undergo conformational changes that involve the disruption of the polar core and three element interaction site, the two main auto-inhibitory intramolecular interactions. This renders the arrestin C-terminus and phosphate-sensing N-domain solvent-exposed, accompanied by a ∼18° interdomain rotation. Especially, since arrestins have no enzymatic function, these conformational changes can be seen as hallmarks of arrestin activation. The release of the arrestin C-terminus is furthermore hypothesized to play a central role in the mediation of arrestin-dependent downstream functions. It harbors binding motifs for the adaptor protein 2 (AP2) complex and clathrin (Goodman et al., 1996; Krupnick et al., 1997; Laporte et al., 2000), in addition to a mitogen-activated protein kinase kinase (MEK) phosphorylation site (Cassier et al., 2017) that enables scaffolding of mitogen-activated protein kinases (MAPKs). Hence, arrestins are able to facilitate clathrin-dependent GPCR internalization and enhance G protein-induced MAPK signaling. In recent years arrestins have been shown to assume distinct conformational states, accommodating not only the active structure of a GPCR but also its specific intracellular phosphorylation. Depending on the overall geometry of the resulting GPCR–arrestin complex, a certain set of effector proteins may then be recruited to orchestrate specific functions.

Crystal structures (Shukla et al., 2013; Kang et al., 2015) and cryo-electron microscopy (Thomsen et al., 2016; Huang et al., 2020; Lee et al., 2020; Staus et al., 2020) studies have shown that these GPCR–arrestin complexes can occur in different configurations. Although they are most probably not mutually exclusive but rather present in a certain equilibrium, different GPCRs make use of distinct binding interfaces when coupling to arrestins. The two main interaction sites on the receptor are constituted by the opened intracellular cavity of the active GPCR and phosphorylated peptide stretches like the C-terminus or intracellular loop 3 (IL3). Arrestins bind to these phosphorylated regions via positive charges buried in their N-domain. Subsequently, the active GPCR cavity is engaged by the arrestin FLR, which is inserted into the receptor transmembrane helix bundle and might assume an alpha-helical structure to stabilize this interaction (Kang et al., 2015). GPCR–arrestin complexes that make use of both binding interfaces were termed either “core,” “tight,” or “snuggly” and are usually characterized by high affinity binding and uncoupling of G proteins. Recently, GPCR–arrestin complexes were discovered that only rely on the interaction between the arrestin N-domain and the phosphorylated GPCR C-terminus (Thomsen et al., 2016; Nguyen et al., 2019). This complex configuration is independent of the FLR and does not utilize the transmembrane helix bundle binding interface, therefore still allowing further activation of G proteins (Figure 1B). Moreover, arrestins that associate with GPCRs in this “hanging” configuration can still assume active conformations and have been shown to functionally increase receptor internalization (Kumari et al., 2017). Thus, GRK-mediated receptor phosphorylation is crucial for the formation of “core” and “hanging” GPCR–arrestin complexes. Phosphorylation is often also hypothesized to be the starting point of arrestin complex formation, however, the precise determination of succession of these binding events is still occluded, as arrestins also have an affinity for active, yet unphosphorylated GPCRs (Gurevich and Gurevich, 2006; Haider et al., 2019; Drube et al., 2021).

**FIGURE 2:**
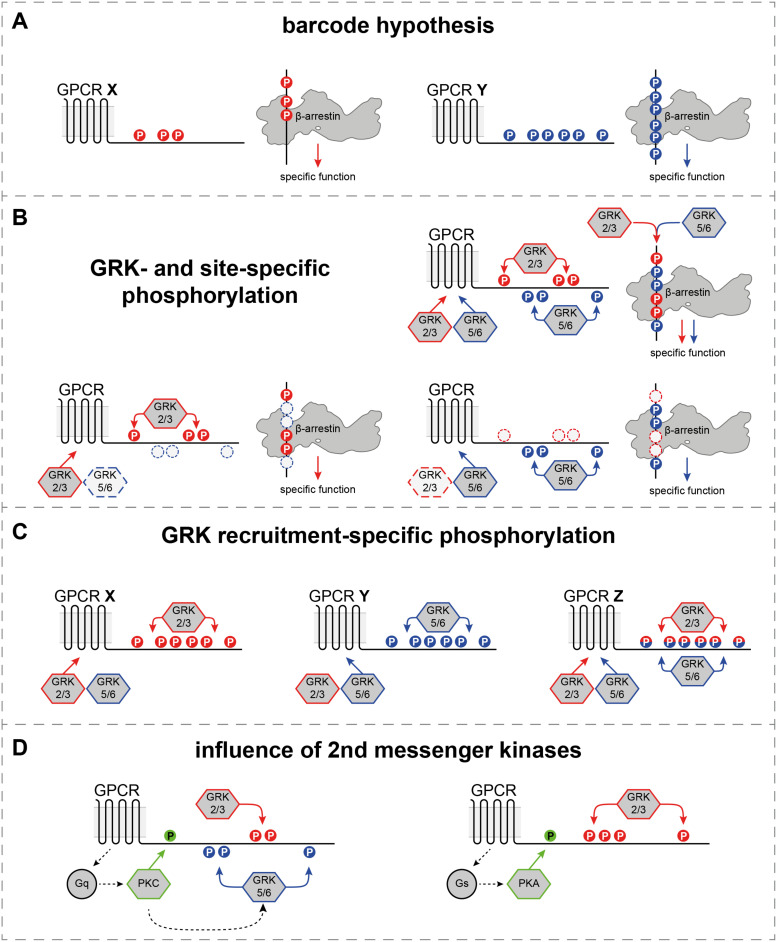
Differential and GRK-specific GPCR phosphorylation induces distinct β-arrestin mediated functions. **(A)** The most straightforward way to interpret the “barcode” hypothesis, as different GPCRs feature different C-terminal phosphorylation patterns to induce distinct β-arrestin functions. **(B)** Individual GRK isoforms or families (GRK2/3 or GRK5/6) have been shown to preferentially phosphorylate specific sites at different GPCR C-termini (Nobles et al., 2011; Doll et al., 2012; Miess et al., 2018). Depending on the availability of kinases in a cellular system, the same GPCR could be phosphorylated by GRK2/3 or GRK5/6 only, to induce specific functions, or by all GRK isoforms to achieve the activation of all possible β-arrestin functions. **(C)** Certain GPCRs have been shown to be functionally phosphorylated by GRK2/3 only, or GRK2/3/5/6 (Drube et al., 2021). This might constitute another layer of coupling preference at the foundation of the “barcode” hypothesis. **(D)** Second messenger kinases, like PKC and PKA are activated by the primary G protein signaling and have been shown to phosphorylate GPCRs directly. Additionally, they are able to modulate the activity of certain GRK isoforms or families (Chuang et al., 1995; Winstel et al., 1996; Pronin and Benovic, 1997).

Differential spacing of negative charges at the receptor C-terminus has been shown to induce specific conformational changes in arrestins (Lee et al., 2016; Nuber et al., 2016; Mayer et al., 2019). Furthermore, these conformational states have been linked with distinct functional outcomes (Yang et al., 2015; Lee et al., 2016). As these findings suggest that every GPCR–arrestin complex is formed in a specific configuration, this could explain how only two β-arrestin isoforms are able to mediate targeted processes for more than 800 different GPCRs. Based on this argumentation, the “barcode” hypothesis was put forward, stating that the arrestin N-domain is capable of recognizing a plethora of different GPCR phosphorylation states. Different phosphorylation patterns (“barcodes”) would then only induce certain conformational changes that dictate arrestin functions for the interaction with a given GPCR (Figure 2A).

## How Arrestins Interpret Different Phosphorylation Patterns: The “Barcode” Hypothesis

In its most straightforward interpretation, the “barcode” hypothesis states that arrestins react to different phosphorylation patterns via specific conformational changes in order to fulfill targeted functions (Figure 2A). This adequately explains how different GPCRs can experience divergent arrestin-mediated regulation, and constitutes a solid foundation for the investigation of these phosphorylation-dependent processes. In line with this hypothesis, arrestins have been shown to undergo specific conformational changes for the coupling with different GPCRs (Lee et al., 2016; Nuber et al., 2016).

Multiple studies showed that different GRK isoforms preferentially phosphorylate specific sites of the same GPCR (Nobles et al., 2011; Doll et al., 2012; Miess et al., 2018). These findings expand the “barcode” hypothesis, as they suggest that one receptor may feature different phosphorylation states depending on the cellular context and the availability of kinases. For example, the β2 adrenergic receptor (β2ADR) has been shown to be differentially phosphorylated by GRK2 or GRK6, resulting in kinase-specific C-terminal phosphorylation patterns (Nobles et al., 2011). From these results, a “site-specific barcode” hypothesis emerged, which suggests that GRK2/3 or GRK5/6 phosphorylate the receptor at different sites to induce divergent functions (Figure 2B). Thus, depending on the available kinases, a GPCR could be phosphorylated at GRK2/3- or GRK5/6-specific sites only, or fully phosphorylated by all four GRK isoforms to induce all possible arrestin-mediated functions.

Indeed, there is evidence that supports this hypothesis as specific phosphorylation patterns have been linked with distinct conformational changes in β-arrestins and downstream functions (Yang et al., 2015). Interestingly, GRK2/3 phosphorylation was proposed to be the driver of receptor internalization, whereas GRK5/6-mediated GPCR phosphorylation was linked with increased ERK signaling (Kim et al., 2005; Ren et al., 2005; Yang et al., 2015). In contrast to these reports, overlapping or even opposing effects for individual GRK isoforms were identified, depending on the used cellular system and the investigated receptor (Tran et al., 2004; Zhu et al., 2013).

The mentioned studies rely on siRNA/shRNA approaches or GRK inhibitors to investigate the impact of individual GRK isoforms on GPCR regulation. These methods bear the risk of co-analyzing a remaining expression of targeted GRK(s) in knockdown approaches, or potential off-target effects of pharmacological intervention. Furthermore, the impact of these methods depends on the initial endogenous GRK expression levels, which were not assessed in these studies. As an example, the knockdown or inhibition of GRK2 would have less pronounced effects in a cellular system that genuinely features a low expression of GRK2. Non-visual GRKs are usually thought of as ubiquitously expressed and their actual tissue distribution is underappreciated in most studies that focus on molecular mechanisms of GPCR regulation. Additionally, no clear consensus sequences have been identified for specific GRK isoforms, although efforts were made to fill this gap (Pinna and Ruzzene, 1996; Asai et al., 2014; Kang et al., 2020).

Recent studies which utilize the CRISPR/Cas9 technology to achieve a partial (Moller et al., 2020) or complete genetic ablation (Drube et al., 2021) of GRK2, 3, 5, and/or 6 suggest that GPCR-specific GRK-coupling preferences might determine which isoforms regulate a given receptor (Figure 2C). Using β-arrestin recruitment as a read-out for GRK-mediated receptor regulation, two subsets of GPCRs have been identified (Drube et al., 2021): receptors that are functionally phosphorylated by GRK2, 3, 5, and 6 and those for which arrestin recruitment could only be mediated by GRK2 and 3. By analysis of the β2ADR, this study shows that even though GRK2 and GRK6 preferentially phosphorylate distinct C-terminal sites (Nobles et al., 2011), the individual overexpression of either kinase mediates β-arrestin recruitment to the same extent. These findings indicate that different GRK isoforms might be able to induce identical GPCR regulation on a molecular level, but specific contributions to these processes are ultimately defined by the relative tissue expression of GRK2, 3, 5, and 6.

GPCR phosphorylation patterns are also influenced by second messenger kinases like protein kinase A (PKA) or protein kinase C (PKC) (Figure 2D). Those kinases are activated via the primary G_s_ or G_q_ signaling pathways, respectively, and have been shown to phosphorylate GPCRs directly. Interestingly, PKC also phosphorylates GRKs and is able to modulate their activity (Chuang et al., 1995; Winstel et al., 1996; Pronin and Benovic, 1997). Thus, the resulting phosphorylation “barcode” of a GPCR might be changed by direct phosphorylation or via increasing or decreasing the activity of specific GRK isoforms, depending on the individual G protein-coupling preference. This cross-talk between GPCR regulating kinases is largely underappreciated in recent literature and needs more elaboration to complete our understanding of phosphorylation-dependent GPCR regulation.

Additionally, there are more unanswered biological questions at the foundation of the “barcode” hypothesis. Given that GRK isoforms preferably phosphorylate different sites, how is it that specific GRK consensus sequences are still elusive? Can a receptor molecule be phosphorylated by more than one GRK? If so, does the sequence in which a GPCR is phosphorylated by multiple GRK isoforms change the resulting phosphorylation pattern? These questions still need to be answered by future experiments in order to unravel the intricate details of GPCR regulation.

## How the “Barcode” Hypothesis Can Be Interpreted Structurally: Intrinsically Disordered Regions

One possible extension to explain the “barcode” hypothesis structurally, which goes beyond pure electrostatic interactions of negatively charged phosphate groups on the receptor with basic amino acid side chains of arrestin, might be intrinsically disordered regions (IDRs) of the GPCR itself. IDRs are longer protein regions which do not show a persistent traditional secondary structure of an α-helix or β-sheet (van der Lee et al., 2014; Shammas et al., 2016). Such disordered regions are frequently found in proteins which are involved in signaling cascades (Wright and Dyson, 2015). Intriguingly, IDRs can form different secondary structures when interacting with specific binding partners. An impressive example is the protein p53 which was crystallized with 14 different binding partners and depending on the complex partner, the IDRs of p53 exhibited very different structures (Oldfield and Dunker, 2014). The analysis of GPCR sequences identified IDRs with >50 amino acids in three major receptor regions, namely the N-terminus, the third intracellular loop (IL3), and the receptor C-terminus (Jaakola et al., 2005; Venkatakrishnan et al., 2014). Not surprisingly, the two intracellular regions are well known to be involved in the signal transduction of GPCRs. Due to their flexibility, they frequently need to be truncated or substituted to increase receptor stability in structural biology approaches (Fonin et al., 2019). Furthermore, IDRs are frequently subject to post-translational modifications which help to support structural stabilization of such regions (Venkatakrishnan et al., 2014). Most commonly IDRs are stabilized by phosphorylation, followed by less common ubiquitination (Bah and Forman-Kay, 2016). Both are well known post-translational modifications for GPCRs occurring within IL3 and the C-terminus of the receptor (Patwardhan et al., 2021). Since IDRs are characterized by a lack of persistent structure (Shammas et al., 2016), their folding state may greatly influence the kinetics of interactions with other partners. For example, increasing the proportion of IDRs with a structure that resembles the bound state might enhance the binding affinity for the partner protein (Shammas et al., 2016). This might be due to effects either on the binding on-rate (k_on_) or off-rate (k_off_) of the complex.

If we now carefully consider these possibilities, we can envision that a given GPCR interacts with a GRK and depending on their relative complex geometry, this event will add the first phosphate group to the receptor stretch which is closest to the active site of the GRK. Early experimental evidence for such a scenario was demonstrated for rhodopsin 30 years ago when even exogenous peptides in the vicinity of GRK1 were phosphorylated (Palczewski et al., 1991). This initial phosphorylation could have local structural consequences and allow or disallow certain residues of the receptor to be phosphorylated next. Depending on the GRK subtype, this can have different consequences for the phosphorylation pattern of a given GPCR. In the case that a GPCR is phosphorylated by more than one GRK, even the relative sequence of GRKs phosphorylating the receptor might have differential consequences. This relative order could be dominated by either different GRK expression levels or accessibility of the GPCR. Such a scenario could help to explain the apparent lack of consensus sequences for GRKs and account for altered GPCR signaling when certain GRKs are up- or downregulated under pathophysiological conditions.

GRKs and β-arrestins are often stated to be ubiquitiously expressed (Nogues et al., 2018). However, a detailed comparative analysis of the tissue and cell-type specific expression pattern of β-arrestins or GRKs is currently not available (Nogues et al., 2018). Therefore, to understand the GRK/arrestin regulatory system in more detail, we analyzed the Human Protein Atlas (HPA)^1^ (Thul et al., 2017) for reported expression levels of GRK2, 3, 5, 6, and the two β-arrestins. Furthermore, we included five human arrestin-domain-containing (ARRDC) proteins, also called α-arrestins, based on similarities in mechanistic substrate recognition (Aubry et al., 2009; Kang et al., 2014): Their yeast homolog proteins named ART (arrestin related trafficking adaptors as synonym for ARRDCs in yeast) are reported to use a basic patch in their arrestin domain to recognize the exposed acidic sorting motive of their substrate, for instance a nutrient transporter. To be recognized, the transporter must exist in a conformation that exposes the acidic sorting motif. This exposure occurs during the substrate transport process (active protein state) and is further assisted by phosphorylation (Kahlhofer et al., 2021). Interestingly, ARRDCs lack the auto-inhibitory polar core region seen in visual and β-arrestins and might therefore resemble more of an active arrestin state. Although little is currently known on the function of human ARRDCs, these proteins were reported to interact with GPCRs (Tian et al., 2016). In combination with the mechanistic similarities from their yeast homologs, this observation encouraged us to assemble the information on ARRDC expression besides the β-arrestins.

## The Tissue Perspective: Are GRK Expression Levels the Key?

To evaluate the composition of GPCR-regulating systems for different tissues, we accessed the HPA and analyzed the relative tissue-specific expression levels for various GRK and arrestin isoforms. The HPA is a largescale project, aiming to elucidate human gene expression and localization in cells, tissues, and organs (Uhlen et al., 2015). Since its first publication in 2005, the website has been updated multiple times to include an increasing amount of data generated by different techniques and to combine information from various sources. To compare expression levels of the four ubiquitously expressed GRK isoforms, the two β-arrestins, and ARRDC1-5, we utilized the consensus transcriptomics data of the HPA, the Genotype-Tissue Expression (GTEx), and the Functional Annotation of Mammalian Genomes 5 (FANTOM5) project, made available on the HPA website^2^ (HPA version 20.1, Ensembl version 92.38, last accessed March 10th, 2021). Although mRNA expression levels do not always equate to protein levels in the cells, we nevertheless assume that the mRNA levels somewhat reflect the resulting protein levels. Therefore, we used the available mRNA expression data for our analysis, as it is more detailed than the existing protein expression data. For each gene, the consensus normalized RNA expression (NX) value is calculated via normalization to the maximum expression value found in the three sources (Table 1). By comparing the consensus NX values, different expression patterns within distinct tissues can be identified.

**TABLE 1:** Relative tissue expression of GRK2, 3, 5, and 6, β-arrestin1 and -2, and ARRDC1-5.

Index	Tissue	GRK2	GRK3	GRK5	GRK6	βarr-1	βarr2	ARRDC1	ARRDC2	ARRDC3	ARRDC4	ARRDC5
1	Adipose tissue	13.1	28.0	15.9	6.8	20.9	21.6	8.4	26.7	40.4	9.2	0.2
2	Adrenal gland	10.7	6.0	8.3	5.5	5.4	15.6	8.9	5.3	37.9	9.3	0.0
3	Amygdala	24.5	11.9	3.4	7.8	27.7	23.7	5.3	10.4	9.3	11.4	0.2
4	Appendix	35.8	12.4	16.8	26.8	14.7	47.8	16.9	12.5	12.3	11.5	0.7
5	B-cells	4.1	6.2	9.3	9.2	0.1	2.2	9.9	5.9	4.7	0.5	2.6
6	Basal ganglia	17.0	10.8	7.8	7.3	31.7	18.3	7.0	34.6	14.6	18.4	0.2
7	Bone marrow	78.2	7.0	2.7	68.1	9.6	102.8	22.1	39.3	68.7	6.7	2.2
8	Breast	17.0	11.3	10.2	10.2	15.9	10.0	10.7	27.7	40.8	14.8	0.2
9	Cerebellum	32.9	7.9	6.5	7.7	24.4	28.1	4.2	3.2	17.9	3.3	0.2
10	Cerebral cortex	35.3	18.9	5.0	10.9	36.7	26.3	8.0	19.5	13.8	16.3	0.6
11	Cervix, uterine	12.8	4.7	12.5	5.9	6.9	8.7	11.1	10.9	25.6	9.4	0.2
12	Colon	17.2	4.0	18.8	9.2	17.8	16.1	21.4	9.6	18.6	27.4	0.2
13	Corpus callosum	12.2	3.1	3.9	6.6	11.2	20.4	6.0	28.3	14.8	67.3	0.2
14	Dendritic cells	6.0	3.7	2.2	5.7	12.7	18.0	32.1	3.1	3.4	2.4	3.9
15	Ductus deferens	12.6	0.7	4.5	5.0	2.0	1.9	23.2	7.3	9.4	11.6	0.2
16	Duodenum	17.5	3.0	12.4	10.9	15.4	23.0	24.2	6.9	4.5	11.2	0.1
17	Endometrium	11.4	5.7	13.4	8.0	7.6	7.4	6.9	7.6	30.0	10.7	0.2
18	Epididymis	9.9	3.1	12.2	6.0	4.5	7.8	12.5	4.7	14.6	11.9	0.2
19	Esophagus	20.2	2.7	8.1	11.0	13.8	7.1	22.4	8.1	28.5	11.8	0.2
20	Fallopian tube	12.6	9.1	7.7	6.6	12.1	10.8	8.5	8.5	13.3	10.8	0.1
21	Gallbladder	17.6	5.0	19.5	9.3	9.2	14.8	20.6	13.1	17.4	14.7	0.3
22	Granulocytes	21.0	5.6	6.5	32.1	14.7	52.9	52.7	3.7	43.1	3.1	3.3
23	Heart muscle	13.6	4.6	45.4	6.2	12.2	10.2	7.1	8.5	16.0	9.7	0.2
24	Hippoc. formation	21.9	15.7	4.2	8.0	25.0	27.3	6.0	14.1	9.5	18.7	0.2
25	Hypothalamus	17.4	8.3	4.2	7.3	16.0	20.9	4.8	8.2	8.6	9.2	0.1
26	Kidney	13.0	3.0	4.3	5.7	9.1	10.5	13.9	10.7	23.5	14.1	0.2
27	Liver	14.1	3.4	5.7	6.6	7.4	15.6	14.6	9.8	33.4	21.8	0.2
28	Lung	19.1	9.4	27.6	11.1	37.3	35.0	19.6	22.6	32.3	14.1	0.5
29	Lymph node	42.8	14.1	8.8	37.6	9.7	31.3	19.3	19.0	14.6	6.4	2.2
30	Midbrain	13.4	9.7	3.7	7.1	19.1	20.1	5.9	47.5	14.6	18.1	0.2
31	Monocytes	18.3	8.0	3.7	22.6	37.5	32.8	33.9	6.3	7.7	5.1	1.2
32	NK-cells	3.8	0.0	0.2	17.4	3.9	5.9	6.6	7.2	5.3	0.1	1.7
33	Olfactory region	19.0	9.8	2.5	10.7	25.7	19.6	7.6	9.0	5.7	15.2	0.2
34	Ovary	9.8	4.1	16.5	6.1	12.5	8.7	4.7	4.9	57.9	7.4	0.2
35	Pancreas	10.4	8.4	3.5	12.1	28.8	8.3	30.4	6.2	14.4	25.0	0.2
36	Parathyroid gland	9.7	4.4	26.2	3.5	1.8	6.6	6.9	18.7	20.5	3.7	0.0
37	Pituitary gland	12.2	8.2	6.3	5.6	3.3	10.2	12.0	5.7	15.4	6.5	0.2
38	Placenta	12.7	4.5	24.0	4.9	17.7	16.0	15.9	8.1	44.7	13.0	0.2
39	Pons and medulla	15.7	9.6	4.5	6.7	21.8	19.7	7.9	20.1	13.4	29.4	0.2
40	Prostate	17.8	8.2	7.5	8.1	12	7.4	16	10.1	26.7	8.3	0.2
41	Rectum	16.2	4.3	6.4	5.5	16.9	13.2	6.4	7.2	12.6	17	0.1
42	Retina	9.3	4.8	5.6	5	10.1	10.7	9.3	6	17.8	4.6	0.2
43	Salivary gland	17.4	3.7	5	10.8	8.5	8.7	22.1	15.3	43.2	6.5	0.2
44	Seminal vesicle	20.8	1.9	6.7	5.2	7.5	6.2	26.7	11.6	18.1	13.5	0.2
45	Skeletal muscle	20.8	1.4	12.8	7.4	7.2	5.5	9.1	60.7	58.4	10.1	0.2
46	Skin	23.5	4.1	4.7	7.7	11.6	5.8	15.2	9.4	24.6	25.3	0
47	Small intestine	26.3	6.1	10.4	14.5	18.5	21	28.4	13.3	10.3	15.3	1
48	Smooth muscle	12.2	6.2	10.8	7.8	15.9	13.7	6.2	6.7	16.6	14	0.2
49	Spinal cord	12.5	4.9	4.8	5.4	6.4	21.7	4.7	16.8	19.9	48.7	0.3
50	Spleen	59.6	23.2	17	34.3	26.5	66.6	21.5	18.7	16	5.9	2.3
51	Stomach	17.4	4	13.9	9.1	23.1	12.5	30.7	12.1	11.7	18.1	0.2
52	T-cells	5.3	0.4	11.3	11.7	3.6	11.4	12.4	16	15.4	0.1	3.5
53	Testis	6.8	14.8	3.5	10.5	3.1	5	6.9	3.2	12.7	10.2	29.1
54	Thalamus	11.1	2.7	4.7	5.1	19	18.3	4.5	32.9	15.2	41.9	0.2
55	Thymus	35.9	11.6	9.8	32.3	3.8	23.5	16.6	10.5	15.6	1.9	0.2
56	Thyroid gland	10.1	4.5	8.9	5.3	6.4	6.4	14.4	14.3	59	14.9	0.2
57	Tongue	13.6	1.3	10.7	6.4	3.9	3.2	11.6	4.9	17.2	4.5	0.2
58	Tonsil	37.5	15.2	8.6	26.5	7.2	17	21.2	19.1	24.1	3.5	0.8
59	Total PBMC	7.4	1.6	4.2	9.1	10.9	16	13.4	4.7	6.4	0.8	0.9
60	Urinary bladder	17.1	6.7	9.5	11.4	13.2	15.7	11.4	9.3	47.7	11.7	0.2
61	Vagina	15.5	2.5	13.6	6.5	7.9	7.1	12.6	11.3	26	12.4	0.2

**Consensus transcriptomics data of the Human Protein Atlas, the Genotype-Tissue Expression, and the Functional Annotation of Mammalian Genomes 5 project as normalized expression (NX) calculated in relation to the maximum NX value in the three sources for each gene. The data are based on the Human Protein Atlas version 20.1 (https://www.proteinatlas.org/), last accessed March 10th, 2021.**

With this approach, we found tissues that predominantly express one GRK, with all other isoforms being comparatively lower expressed [e.g., GRK5 in heart muscle (23) or GRK2 in skin (46)]. The database also reveals tissues in which two GRK isoforms are comparably high expressed [e.g., GRK2 and 6 in bone marrow (7) or GRK2 and 5 in gallbladder (21)] or tissues with similar NX values for all GRKs [e.g., smooth muscle (48)]. Interestingly, some functional groups of tissues, categorized according to the HPA, share common expression patterns. For example, GRK2 is the predominant isoform expressed in all assessed tissues of the brain, whereas GRK3 is the most abundant isoform in adipose tissue. Bone marrow and lymphoid tissues feature high expression levels of GRK2 and GRK6. Furthermore, GRK6 is highly expressed in all assessed blood cells. Some of them express GRK6 and GRK2 at similar levels [dendritic cells (14) and total peripheral blood mononuclear cells (PBMC; 59)], or feature GRK2 as the second highest expressed isoform [e.g., granulocytes (22) and monocytes (31)]. In contrast, B- (5) and T-cells (52) show similar expression levels of GRK6 and GRK5.

**FIGURE 3:**
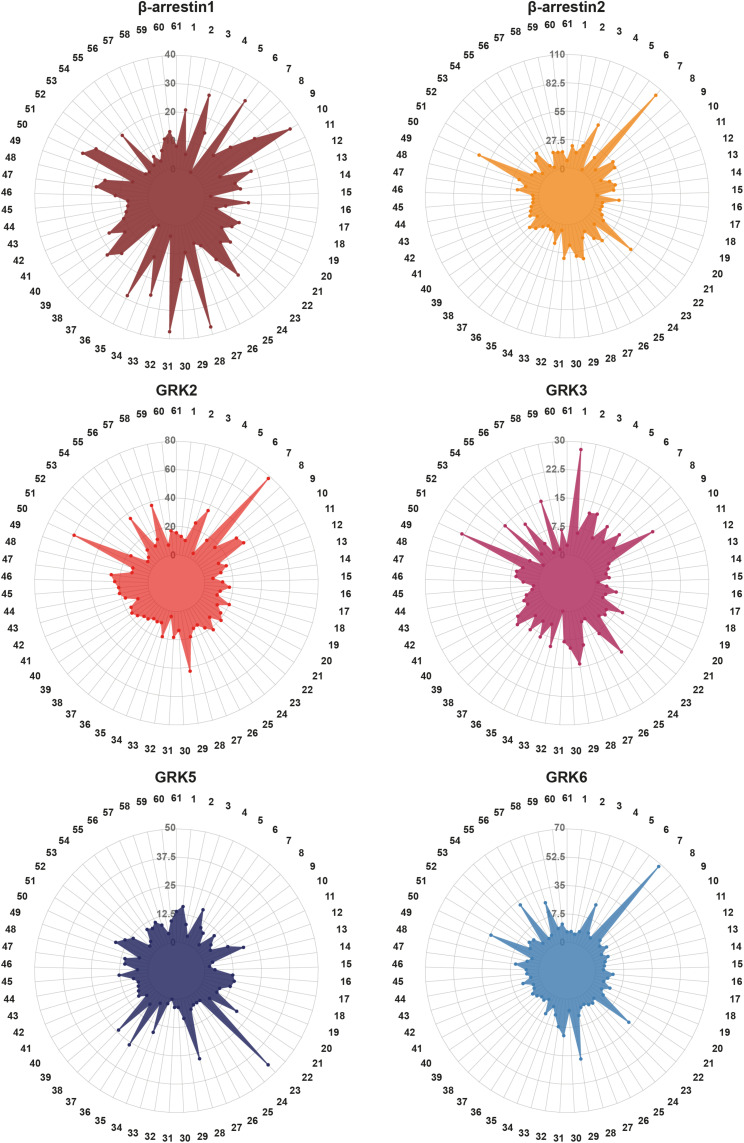
Visualization of the tissue-specific expression levels of GRK2, 3, 5, and 6, β-arrestin1 and -2. The consensus expression data of Table 1 are visualized in radar plots for each protein in 61 tissues. The numbers correspond to the assigned indices of tissues in Table 1. The data are based on the Human Protein Atlas version 20.1 (https://www.proteinatlas.org/), last accessed March 10th, 2021.

**FIGURE 4:**
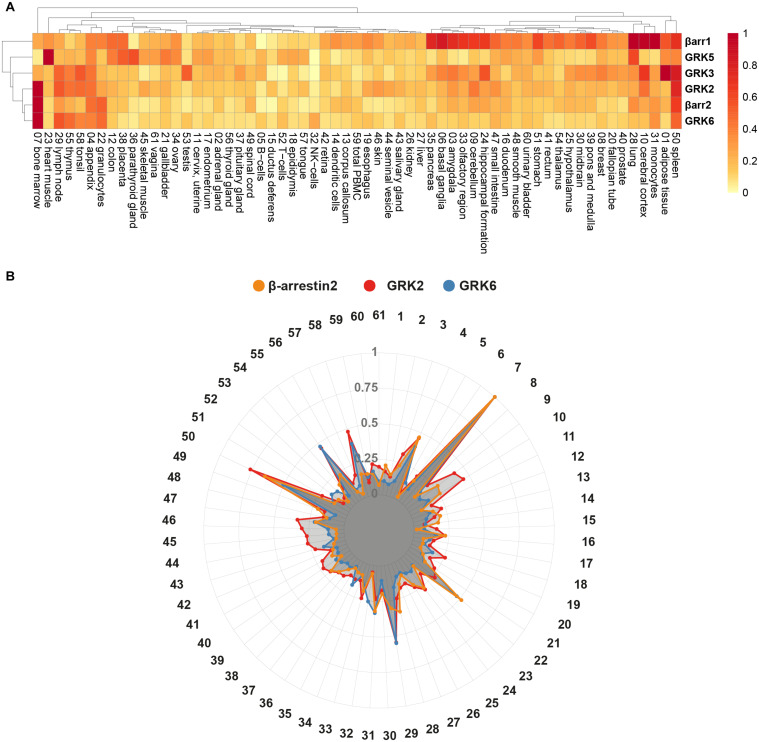
Clustering of relative protein expression and overlay of GRK2, GRK6, and β-arrestin2 tissue expression. **(A)** Clustering of relative expression profiles of GRK2, 3, 5, 6, and β-arrestin1 and 2, according to Euclidean distance. The NX values of Table 1 were normalized to the respective maximal tissue expression for each protein. The clustering heatmap was generated using the *pheatmap* R package (Kolde (2013). pheatmap: Pretty Heatmaps. R package version 1.0.12, http://CRAN.R-project.org/package=pheatmap). **(B)** Relative tissue expression of GRK2 (red), GRK6 (blue), and β-arrestin2 (yellow) are shown together. The data are based on the Human Protein Atlas version 20.1 (https://www.proteinatlas.org/), last accessed March 10th, 2021.

These different GRK expression patterns occur alongside distinct expression levels of β-arrestins. Some tissues express the two β-arrestin isoforms at similar levels [e.g., colon (12) or lung (28)], while other tissues feature a predominant expression of one isoform [e.g., β-arrestin2 in bone marrow (7) or β-arrestin1 in pancreas (35)]. Considering the expression levels of ARRDC1-5 adds another layer of complexity to this system of GRK-mediated GPCR regulation. To visualize the respective protein-specific expression profiles for all listed tissues in the HPA, we prepared radar plots of β-arrestin1 and 2, GRK2, 3, 5, and 6 (Figure 3). Using a clustering heatmap [generated with R package *pheatmap* (Kolde, 2013. pheatmap: Pretty Heatmaps. R package version 1.0.12^3^)], we analyzed the relative expression of these genes, normalized to the respective maximal expression (Figure 4A). The clustering algorithm identified the highest degree of similarity for the relative expression profiles of GRK2, 6, and β-arrestin2, according to the Euclidean distance. Following this analysis, we depicted the relative expression data for these three genes as an overlay radar chart (Figure 4B). This overlay reveals stunningly similar tissue expression patterns for these three proteins. It is tempting to speculate that GRK2, 6, and β-arrestin2 constitute an intricate system, in which disbalance is unfavorable and might lead to dysfunctional GPCR regulation under pathological conditions.

## Pathophysiological Effects of Dysregulated GRK Expression Changes

As every cell of the human body expresses GPCRs, the regulated expression levels of GRKs and β-arrestins are crucial to maintain healthy cellular and organ functions. In the following section, we highlight selected examples where a dysregulation of this delicate regulatory system might contribute to the development or progress of different pathological conditions.

The role of dysregulated GRK expression in the development of tumors was subject to extensive work and we refer to excellent reviews for further reading (Nogues et al., 2017; Nogues et al., 2018; Sun et al., 2018; Yu et al., 2018). All non-visual GRKs have been found to be dysregulated in at least one tumor model where they can act either as oncogenes or as tumor suppressors. As one example, the mean mRNA expression level of GRK5 for all analyzed tissues (Figure 3 and Table 1) is 9.7 NX. In brain tissues (indices 3, 6, 9, 10, 13, 24, 25, 30, 33, 39, 49, and 54 of Table 1), the expression levels of GRK5 range from 2.5 to 7.8 NX. Similarly low expression levels are also seen in prostate (Table 1, index 40) with a relative expression of 7.5 NX. In these naturally low GRK5 expressing tissues, upregulation of GRK5 is increasing aggressiveness of glioma (Kaur et al., 2013) and is associated with increased proliferation of prostate cancer (Kim et al., 2012; Chakraborty et al., 2014; DeRita et al., 2017). In contrast, downregulation of GRK5 expression in colon (Table 1, index 12), a tissue with a high expression of 18.8 NX, leads to promoted proliferation in colorectal cancer (Wu et al., 2011). GRKs cannot be generally classified as either tumor suppressors or promotors, since their influence on tumor progression is highly specific for individual cancer entities or tested cell lines (Sun et al., 2018). It is tempting to speculate that upregulation of GRK levels in tissues that naturally feature a low expression level of that given GRK, or downregulation of GRK levels in high expressing tissues might allow a prediction of the impact on cancer progression. This again strengthens the idea that the balance of different players in the given cellular context is the key for physiological regulation of cell growth.

GRKs are important regulators of cell migration, which is crucial for the formation of metastases. Hence, dysregulated GRK levels influence the migratory potential of cancer cells. Changes in GRK2 expression lead to different outcomes depending on the used stimuli and cell type and were extensively discussed elsewhere (Penela et al., 2014). Again, a general association of up- or downregulation of GRK2 with reduction or promotion of migration cannot be made. GRK3 regulates CXCR4-mediated migration and metastasis in breast cancer cell models (Billard et al., 2016). It was shown that shRNA mediated downregulation of GRK3 in breast cancer cell lines led to an increased migration toward CXCL12, whereas overexpression of GRK3 diminished the chemotaxis.

A study using GRK6 knockout (GRK6^–/–^) mice showed that the absence of GRK6 led to increased growth of subcutaneously injected Lewis lung cancer cells, and an increased formation of metastases formed by tail vain injected Lewis lung cancer cells (Raghuwanshi et al., 2013). In this model, CXCR2-mediated promotion of metastasis is regulated by GRK6, and the loss of this negative regulator promotes the malignant phenotype.

Besides the involvement of GRKs in cancer biology, the role of GRK2 in the cardiovascular system is also well studied (Huang et al., 2011; Schumacher and Koch, 2017; Murga et al., 2019). The importance of GRK2 in the heart is highlighted by the fact, that homozygous GRK2^–/–^ mouse embryos exhibited a more than 70% decreased cardiac ejection fraction (Jaber et al., 1996), whereas heterozygous GRK2^+ ⁣/−^ mice showed increased contractile function compared to wild type mice (Huang et al., 2011). This again indicates that the balanced expression is important for physiological function of GRKs and that any change in this delicate system often lead to unpredictable outcomes.

Besides adaptive dysregulation by pathophysiological conditions, gene mutations can also lead to altered expression levels. Mutations in GRK2 were detected in patients suffering from Jeune syndrome (Bosakova et al., 2020). In one patient a mutation was identified to cause a functional loss of GRK2. Interestingly, this did not lead to expected embryonic lethality as seen in mice (Jaber et al., 1996), as the patient was born alive, but passed away 5 days after birth. GRK2 was identified as an essential regulator of skeletogenesis (Bosakova et al., 2020). The patient had a very small chest and suffered from pulmonary insufficiency, but did not show gross abnormalities in the central nervous system. Functional analyses in the same study revealed an impairment of Hedgehog and canonical Wnt signaling leading to the observed phenotype.

All described examples so far pointed out the importance to maintain physiological GRK expression levels to prevent pathophysiological conditions. Although this is not the immediate focus of this review, the GPCR–GRK–β-arrestin system is also influenced by changes of β-arrestin expression levels. In 60% of patients suffering from Sezary Syndrome (a rare cutaneous T cell lymphoma), a mono-allelic loss of the β-arrestin2 gene was found (Cristofoletti et al., 2019). Cell culture experiments showed that downregulation of β-arrestin2 led to an impaired internalization of CXCR4 after CXCL12 stimulation, and it was hypothesized that this would lead to an increased migration toward high CXCL12 levels in skin. Another study found that, β-arrestin2 deficiency in dendritic cells promotes migration and cytokine production which contributes to autoimmune encephalomyelitis (Cai et al., 2019). The dysregulated expression of β-arrestin1 was found to be important in context of maternal-fetal tolerance in human pregnancies (Liu et al., 2021) where a strongly reduced mRNA expression of β-arrestin1 was found in villous samples of missed abortion.

## Concluding Remarks

Taken together, there is surmounting evidence that the expression levels of GRKs, arrestins, and GPCRs play a crucial role in the development of pathological conditions. Literature suggests that the regulatory system of GPCRs is a common, yet fine-tuned machinery which is vital for the maintenance of healthy cellular functions. As different tissues express specific sets of GPCRs to properly react to extracellular stimuli, this regulatory system is adjusted via differential expression of GRKs and arrestins to service this exact set of GPCRs. Disturbance of this equilibrated regulation can then have differential consequences, especially considering that malignancies can also feature the overexpression or downregulation of GPCRs. This is highlighted by the seemingly unpredictable behavior of key players, as in cancer, they can act as both, tumor suppressors or oncogenes, depending on the pathological and cellular context. More work has to be done on mapping functional sets of GPCRs expressed by a given cell and understanding the individual impact of different GRK isoforms on their regulation. This tissue-specific point of view, in combination with further development and elaboration of the “barcode” hypothesis might be the key to unraveling the intricate details of GPCR regulation.

## Author Contributions

EM compiled and visualized the tissue expression data. RH and EM illustrated all figures. EM, RH, CH, and JD wrote the manuscript. All authors contributed to the article and approved the submitted version.

## Conflict of Interest

The authors declare that the research was conducted in the absence of any commercial or financial relationships that could be construed as a potential conflict of interest.

## Footnotes

1 http://proteinatlas.org/humanproteome/cell

2 https://www.proteinatlas.org/

3 http://CRAN.R-project.org/package=pheatmap

## Abbreviations

AMP: adenosine monophosphate
AP2: adaptor protein 2
ARRDC, arrestin-domain-containing proteins: α-arrestins
ART, arrestin-related trafficking adaptors: α-arrestins
β 2ADR: β 2 adrenergic receptor
CXCL12, C-X-C motif chemokine 12: also known as stromal cell-derived factor 1 (SDF1)
CXCR2: C-X-C chemokine receptor type 2 also known as Interleukin 8 receptor beta, IL8RB CD182
CXCR4: C-X-C chemokine receptor type 4 also known as fusin or CD184
DAG: diacylglycerol
FANTOM5: Functional Annotation of Mammalian Genomes 5
FLR: finger loop region
GDP: guanosine diphosphate
GPCR: G protein-coupled receptor
GRK: GPCR kinase
GTEx: Genotype-Tissue Expression
GTP: guanosine triphosphate
HPA: Human Protein Atlas
IDR: intrinsically disordered regions
IL3: intracellular loop 3
MAPK: mitogen-activated protein kinase
MEK: mitogen-activated protein kinase kinase
NX: normalized expression
PBMC: peripheral blood mononuclear cells
PDB: Protein Data Bank
PKA: protein kinase A
PKC: protein kinase C
shRNA: short hairpin RNA
siRNA: short interfering RNA.

## References

[B1] Asai D. Toita R. Murata M. Katayama Y. Nakashima H. Kang J. H. (2014). Peptide substrates for G protein-coupled receptor kinase 2. *FEBS Lett.* 588 2129–2132. 10.1016/j.febslet.2014.04.038 24813628

[B2] Aubry L. Guetta D. Klein G. (2009). The arrestin fold: variations on a theme. *Curr. Genom.* 10 133–142. 10.2174/138920209787847014 19794886 PMC2699828

[B3] Bah A. Forman-Kay J. D. (2016). Modulation of intrinsically disordered protein function by post-translational modifications. *J. Biol. Chem.* 291 6696–6705. 10.1074/jbc.R115.695056 26851279 PMC4807257

[B4] Billard M. J. Fitzhugh D. J. Parker J. S. Brozowski J. M. McGinnis M. W. Timoshchenko R. G. (2016). G protein coupled receptor kinase 3 regulates breast cancer migration, invasion, and metastasis. *PLoS One* 11:e0152856. 10.1371/journal.pone.0152856 27049755 PMC4822790

[B5] Bosakova M. Abraham S. P. Nita A. Hruba E. Buchtova M. Taylor S. P. (2020). Mutations in GRK2 cause Jeune syndrome by impairing Hedgehog and canonical Wnt signaling. *EMBO Mol. Med.* 12 e11739. 10.15252/emmm.201911739 33200460 PMC7645380

[B6] Cai Y. Yang C. Yu X. Qian J. Dai M. Wang Y. (2019). Deficiency of beta-Arrestin 2 in dendritic cells contributes to autoimmune diseases. *J. Immunol.* 202 407–420. 10.4049/jimmunol.1800261 30541881

[B7] Cassier E. Gallay N. Bourquard T. Claeysen S. Bockaert J. Crepieux P. (2017). Phosphorylation of beta-arrestin2 at Thr(383) by MEK underlies beta-arrestin-dependent activation of Erk1/2 by GPCRs. *Elife* 6:e23777. 10.7554/eLife.23777 28169830 PMC5325621

[B8] Cato M. C. Yen Y. C. Francis C. J. Elkins K. E. Shareef A. Sterne-Marr R. (2021). The open question of how GPCRs interact with GPCR kinases (GRKs). *Biomolecules* 11:447. 10.3390/biom11030447 33802765 PMC8002388

[B9] Chakraborty P. K. Zhang Y. Coomes A. S. Kim W. J. Stupay R. Lynch L. D. (2014). G protein-coupled receptor kinase GRK5 phosphorylates moesin and regulates metastasis in prostate cancer. *Cancer Res.* 74 3489–3500. 10.1158/0008-5472.CAN-13-2708 24755472

[B10] Chen C. Y. Dion S. B. Kim C. M. Benovic J. L. (1993). Beta-adrenergic receptor kinase. agonist-dependent receptor binding promotes kinase activation. *J. Biol. Chem.* 268 7825–7831. 8096517

[B11] Chuang T. T. LeVine H. III De Blasi A. (1995). Phosphorylation and activation of beta-adrenergic receptor kinase by protein kinase C. *J. Biol. Chem.* 270 18660–18665. 10.1074/jbc.270.31.18660 7629197

[B12] Claing A. Laporte S. A. Caron M. G. Lefkowitz R. J. (2002). Endocytosis of G protein-coupled receptors: roles of G protein-coupled receptor kinases and beta-arrestin proteins. *Prog. Neurobiol.* 66 61–79. 10.1016/s0301-0082(01)00023-5 11900882

[B13] Crepieux P. Poupon A. Langonne-Gallay N. Reiter E. Delgado J. Schaefer M. H. (2017). A comprehensive view of the beta-arrestinome. *Front. Endocrinol.* 8:32. 10.3389/fendo.2017.00032 28321204 PMC5337525

[B14] Cristofoletti C. Bresin A. Caprini E. Russo G. Narducci M. G. (2019). Loss of beta-arrestin-2 gene and possible functional consequences on Sezary Syndrome. *Cell Cycle* 18 1292–1294. 10.1080/15384101.2019.1617007 31106661 PMC6592223

[B15] DeRita R. M. Zerlanko B. Singh A. Lu H. Iozzo R. V. Benovic J. L. (2017). c-Src, insulin-like growth factor I Receptor, G-protein-coupled receptor kinases and focal adhesion kinase are enriched into prostate cancer cell exosomes. *J. Cell Biochem.* 118 66–73. 10.1002/jcb.25611 27232975 PMC5552241

[B16] DeWire S. M. Ahn S. Lefkowitz R. J. Shenoy S. K. (2007). Beta-arrestins and cell signaling. *Annu. Rev. Physiol.* 69 483–510. 10.1146/annurev.physiol.69.022405.154749 17305471

[B17] Doll C. Poll F. Peuker K. Loktev A. Gluck L. Schulz S. (2012). Deciphering micro-opioid receptor phosphorylation and dephosphorylation in HEK293 cells. *Br. J. Pharmacol.* 167 1259–1270. 10.1111/j.1476-5381.2012.02080.x 22725608 PMC3504992

[B18] Drube J. Haider R. S. Matthees E. S. F. Reichel M. Zeiner J. Fritzwanker S. (2021). GRK2/3/5/6 knockout: the impact of individual GRKs on arrestin-binding and GPCR regulation. *bioRxiv* [Preprint]. 10.1101/2021.02.12.430971 PMC8795447 35087057

[B19] Flock T. Hauser A. S. Lund N. Gloriam D. E. Balaji S. Babu M. M. (2017). Selectivity determinants of GPCR-G-protein binding. *Nature* 545 317–322. 10.1038/nature22070 28489817 PMC5846738

[B20] Flock T. Ravarani C. N. J. Sun D. Venkatakrishnan A. J. Kayikci M. Tate C. G. (2015). Universal allosteric mechanism for Galpha activation by GPCRs. *Nature* 524 173–179. 10.1038/nature14663 26147082 PMC4866443

[B21] Fonin A. V. Darling A. L. Kuznetsova I. M. Turoverov K. K. Uversky V. N. (2019). Multi-functionality of proteins involved in GPCR and G protein signaling: making sense of structure-function continuum with intrinsic disorder-based proteoforms. *Cell Mol. Life Sci.* 76 4461–4492. 10.1007/s00018-019-03276-1 31428838 PMC11105632

[B22] Godbole A. Lyga S. Lohse M. J. Calebiro D. (2017). Internalized TSH receptors en route to the TGN induce local Gs-protein signaling and gene transcription. *Nat. Commun.* 8:443. 10.1038/s41467-017-00357-2 28874659 PMC5585343

[B23] Goodman O. B. Jr. Krupnick J. G. Santini F. Gurevich V. V. Penn R. B. Gagnon A. W. (1996). Beta-arrestin acts as a clathrin adaptor in endocytosis of the beta2-adrenergic receptor. *Nature* 383 447–450. 10.1038/383447a0 8837779

[B24] Gurevich E. V. Tesmer J. J. Mushegian A. Gurevich V. V. (2012). G protein-coupled receptor kinases: more than just kinases and not only for GPCRs. *Pharmacol. Ther.* 133 40–69. 10.1016/j.pharmthera.2011.08.001 21903131 PMC3241883

[B25] Gurevich V. V. Gurevich E. V. (2006). The structural basis of arrestin-mediated regulation of G-protein-coupled receptors. *Pharmacol. Ther.* 110 465–502. 10.1016/j.pharmthera.2005.09.008 16460808 PMC2562282

[B26] Gurevich V. V. Gurevich E. V. (2019). GPCR signaling regulation: the role of GRKs and arrestins. *Front. Pharmacol.* 10:125. 10.3389/fphar.2019.00125 30837883 PMC6389790

[B27] Haider R. S. Wilhelm F. Rizk A. Mutt E. Deupi X. Peterhans C. (2019). Arrestin-1 engineering facilitates complex stabilization with native rhodopsin. *Sci. Rep.* 9:439. 10.1038/s41598-018-36881-4 30679635 PMC6346018

[B28] Homan K. T. Tesmer J. J. (2014). Structural insights into G protein-coupled receptor kinase function. *Curr. Opin. Cell Biol.* 27 25–31. 10.1016/j.ceb.2013.10.009 24680427 PMC3971390

[B29] Huang C. C. Tesmer J. J. G. (2011). Recognition in the face of diversity: interactions of heterotrimeric G proteins and G protein-coupled receptor (GPCR) kinases with activated GPCRs. *J. Biol. Chem.* 286 7715–7721. 10.1074/jbc.R109.051847 21199869 PMC3048657

[B30] Huang W. Masureel M. Qu Q. Janetzko J. Inoue A. Kato H. E. (2020). Structure of the neurotensin receptor 1 in complex with beta-arrestin 1. *Nature* 579 303–308. 10.1038/s41586-020-1953-1 31945771 PMC7100716

[B31] Huang Z. M. Gold J. I. Koch W. J. (2011). G protein-coupled receptor kinases in normal and failing myocardium. *Front. Biosci.* 16:3047–3060. 10.2741/3898 21622221 PMC4149910

[B32] Inoue A. Raimondi F. Kadji F. M. N. Singh G. Kishi T. Uwamizu A. (2019). Illuminating G-protein-coupling selectivity of GPCRs. *Cell* 177 1933–1947.e25. 10.1016/j.cell.2019.04.044 31160049 PMC6773469

[B33] Jaakola V. P. Prilusky J. Sussman J. L. Goldman A. (2005). G protein-coupled receptors show unusual patterns of intrinsic unfolding. *Protein Eng. Des. Sel.* 18 103–110. 10.1093/protein/gzi004 15790574

[B34] Jaber M. Koch W. J. Rockman H. Smith B. Bond R. A. Sulik K. K. (1996). Essential role of beta-adrenergic receptor kinase 1 in cardiac development and function. *Proc. Natl. Acad. Sci. U.S.A.* 93 12974–12979. 10.1073/pnas.93.23.12974 8917529 PMC24031

[B35] Kahlhofer J. Leon S. Teis D. Schmidt O. (2021). The alpha-arrestin family of ubiquitin ligase adaptors links metabolism with selective endocytosis. *Biol. Cell* 113 183–219. 10.1111/boc.202000137 33314196

[B36] Kang D. S. Tian X. Benovic J. L. (2014). Role of beta-arrestins and arrestin domain-containing proteins in G protein-coupled receptor trafficking. *Curr. Opin. Cell Biol.* 27 63–71. 10.1016/j.ceb.2013.11.005 24680432 PMC3971387

[B37] Kang J. H. Toita R. Kawano T. Murata M. Asai D. (2020). Design of substrates and inhibitors of G protein-coupled receptor kinase 2 (GRK2) based on its phosphorylation reaction. *Amino Acids* 52 863–870. 10.1007/s00726-020-02864-x 32577910

[B38] Kang Y. Zhou X. E. Gao X. He Y. Liu W. Ishchenko A. (2015). Crystal structure of rhodopsin bound to arrestin by femtosecond X-ray laser. *Nature* 523 561–567. 10.1038/nature14656 26200343 PMC4521999

[B39] Kaur G. Kim J. Kaur R. Tan I. Bloch O. Sun M. Z. (2013). G-protein coupled receptor kinase (GRK)-5 regulates proliferation of glioblastoma-derived stem cells. *J. Clin. Neurosci.* 20 1014–1018. 10.1016/j.jocn.2012.10.008 23693024

[B40] Kim J. I. Chakraborty P. Wang Z. Daaka Y. (2012). G-protein coupled receptor kinase 5 regulates prostate tumor growth. *J. Urol.* 187 322–329. 10.1016/j.juro.2011.09.049 22099983

[B41] Kim J. Ahn S. Ren X. R. Whalen E. J. Reiter E. Wei H. (2005). Functional antagonism of different G protein-coupled receptor kinases for beta-arrestin-mediated angiotensin II receptor signaling. *Proc. Natl. Acad. Sci. U.S.A.* 102 1442–1447. 10.1073/pnas.0409532102 15671181 PMC547874

[B42] Kolde R. (2013.) Available online at: https://cran.r-project.org/web/packages/pheatmap/index.html

[B43] Krupnick J. G. Goodman O. B. Jr. Keen J. H. Benovic J. L. (1997). Arrestin/clathrin interaction. localization of the clathrin binding domain of nonvisual arrestins to the carboxy terminus. *J. Biol. Chem.* 272 15011–15016. 10.1074/jbc.272.23.15011 9169476

[B44] Kumari P. Srivastava A. Ghosh E. Ranjan R. Dogra S. Yadav P. N. (2017). Core engagement with beta-arrestin is dispensable for agonist-induced vasopressin receptor endocytosis and ERK activation. *Mol. Biol. Cell* 28 1003–1010. 10.1091/mbc.E16-12-0818 28228552 PMC5391177

[B45] Laporte S. A. Oakley R. H. Holt J. A. Barak L. S. Caron M. G. (2000). The interaction of beta-arrestin with the AP-2 adaptor is required for the clustering of beta 2-adrenergic receptor into clathrin-coated pits. *J. Biol. Chem.* 275 23120–23126. 10.1074/jbc.M002581200 10770944

[B46] Latorraca N. R. Venkatakrishnan A. J. Dror R. O. (2017). GPCR dynamics: structures in motion. *Chem. Rev.* 117 139–155. 10.1021/acs.chemrev.6b00177 27622975

[B47] Lee M. H. Appleton K. M. Strungs E. G. Kwon J. Y. Morinelli T. A. Peterson Y. K. (2016). The conformational signature of beta-arrestin2 predicts its trafficking and signalling functions. *Nature* 531 665–668. 10.1038/nature17154 27007854 PMC4973468

[B48] Lee Y. Warne T. Nehme R. Pandey S. Dwivedi-Agnihotri H. Chaturvedi M. (2020). Molecular basis of beta-arrestin coupling to formoterol-bound beta1-adrenoceptor. *Nature* 583 862–866. 10.1038/s41586-020-2419-1 32555462 PMC7115876

[B49] Liu T. Ma Y. Yin Q. Zhou H. Fang Y. (2021). Association of beta-arrestin1 and p53-Mdm2 signaling in the development of missed abortion. *J. Obstet. Gynaecol. Res.* 47, 1675–1685. 10.1111/jog.14643, 33611816

[B50] Marinissen M. J. Gutkind J. S. (2001). G-protein-coupled receptors and signaling networks: emerging paradigms. *Trends Pharmacol. Sci.* 22 368–376. 10.1016/s0165-6147(00)01678-3 11431032

[B51] Mayer D. Damberger F. F. Samarasimhareddy M. Feldmueller M. Vuckovic Z. Flock T. (2019). Distinct G protein-coupled receptor phosphorylation motifs modulate arrestin affinity and activation and global conformation. *Nat. Commun.* 10:1261. 10.1038/s41467-019-09204-y 30890705 PMC6424980

[B52] McCarthy N. E. Akhtar M. (2002). Activation of rhodopsin kinase. *Biochem. J.* 363(Pt. 2), 359–364. 10.1042/0264-6021:3630359 11931666 PMC1222487

[B53] Miess E. Gondin A. B. Yousuf A. Steinborn R. Mosslein N. Yang Y. (2018). Multisite phosphorylation is required for sustained interaction with GRKs and arrestins during rapid mu-opioid receptor desensitization. *Sci. Signal.* 11:eaas9609. 10.1126/scisignal.aas9609 30018083

[B54] Milligan G. Kostenis E. (2006). Heterotrimeric G-proteins: a short history. *Br. J. Pharmacol.* 147(Suppl. 1), S46–S55. 10.1038/sj.bjp.0706405 16402120 PMC1760735

[B55] Moller T. C. Pedersen M. F. van Senten J. R. Seiersen S. D. Mathiesen J. M. Bouvier M. (2020). Dissecting the roles of GRK2 and GRK3 in mu-opioid receptor internalization and beta-arrestin2 recruitment using CRISPR/Cas9-edited HEK293 cells. *Sci. Rep.* 10:17395. 10.1038/s41598-020-73674-0 33060647 PMC7567791

[B56] Murga C. Arcones A. C. Cruces-Sande M. Briones A. M. Salaices M. Mayor F. Jr. (2019). G protein-coupled receptor kinase 2 (GRK2) as a potential therapeutic target in cardiovascular and metabolic diseases. *Front. Pharmacol.* 10:112. 10.3389/fphar.2019.00112 30837878 PMC6390810

[B57] Mushegian A. Gurevich V. V. Gurevich E. V. (2012). The origin and evolution of G protein-coupled receptor kinases. *PLoS One* 7:e33806. 10.1371/journal.pone.0033806 22442725 PMC3307776

[B58] Nguyen A. H. Thomsen A. R. B. Cahill T. J. III Huang R. Huang L. Y. Marcink T. (2019). Structure of an endosomal signaling GPCR-G protein-beta-arrestin megacomplex. *Nat. Struct. Mol. Biol.* 26 1123–1131. 10.1038/s41594-019-0330-y 31740855 PMC7108872

[B59] Nobles K. N. Xiao K. Ahn S. Shukla A. K. Lam C. M. Rajagopal S. (2011). Distinct phosphorylation sites on the beta(2)-adrenergic receptor establish a barcode that encodes differential functions of beta-arrestin. *Sci. Signal.* 4:ra51. 10.1126/scisignal.2001707 21868357 PMC3415961

[B60] Nogues L. Palacios-Garcia J. Reglero C. Rivas V. Neves M. Ribas C. (2018). G protein-coupled receptor kinases (GRKs) in tumorigenesis and cancer progression: GPCR regulators and signaling hubs. *Semin. Cancer Biol.* 48 78–90. 10.1016/j.semcancer.2017.04.013 28473253

[B61] Nogues L. Reglero C. Rivas V. Neves M. Penela P. Mayor F. Jr. (2017). G-protein-coupled receptor kinase 2 as a potential modulator of the hallmarks of cancer. *Mol. Pharmacol.* 91 220–228. 10.1124/mol.116.107185 27895163

[B62] Nuber S. Zabel U. Lorenz K. Nuber A. Milligan G. Tobin A. B. (2016). beta-Arrestin biosensors reveal a rapid, receptor-dependent activation/deactivation cycle. *Nature* 531 661–664. 10.1038/nature17198 27007855 PMC5157050

[B63] Nygaard R. Zou Y. Dror R. O. Mildorf T. J. Arlow D. H. Manglik A. (2013). The dynamic process of beta(2)-adrenergic receptor activation. *Cell* 152 532–542. 10.1016/j.cell.2013.01.008 23374348 PMC3586676

[B64] Oldfield C. J. Dunker A. K. (2014). Intrinsically disordered proteins and intrinsically disordered protein regions. *Annu. Rev. Biochem.* 83 553–584. 10.1146/annurev-biochem-072711-164947 24606139

[B65] Oldham W. M. Hamm H. E. (2008). Heterotrimeric G protein activation by G-protein-coupled receptors. *Nat. Rev. Mol. Cell Biol.* 9 60–71. 10.1038/nrm2299 18043707

[B66] Palczewski K. Buczylko J. Kaplan M. W. Polans A. S. Crabb J. W. (1991). Mechanism of rhodopsin kinase activation. *J. Biol. Chem.* 266 12949–12955. 2071581

[B67] Patwardhan A. Cheng N. Trejo J. (2021). Post-translational modifications of G protein-coupled receptors control cellular signaling dynamics in space and time. *Pharmacol. Rev.* 73 120–151. 10.1124/pharmrev.120.000082 33268549 PMC7736832

[B68] Penela P. Nogues L. Mayor F. Jr. (2014). Role of G protein-coupled receptor kinases in cell migration. *Curr. Opin. Cell Biol.* 27 10–17. 10.1016/j.ceb.2013.10.005 24680425

[B69] Pinna L. A. Ruzzene M. (1996). How do protein kinases recognize their substrates? *Biochim. Biophys. Acta* 1314 191–225. 10.1016/s0167-4889(96)00083-3 8982275

[B70] Pronin A. N. Benovic J. L. (1997). Regulation of the G protein-coupled receptor kinase GRK5 by protein kinase C. *J. Biol. Chem.* 272 3806–3812. 10.1074/jbc.272.6.3806 9013639

[B71] Raghuwanshi S. K. Smith N. Rivers E. J. Thomas A. J. Sutton N. Hu Y. (2013). G protein-coupled receptor kinase 6 deficiency promotes angiogenesis, tumor progression, and metastasis. *J. Immunol.* 190 5329–5336. 10.4049/jimmunol.1202058 23589623 PMC3646980

[B72] Ren X. R. Reiter E. Ahn S. Kim J. Chen W. Lefkowitz R. J. (2005). Different G protein-coupled receptor kinases govern G protein and beta-arrestin-mediated signaling of V2 vasopressin receptor. *Proc. Natl. Acad. Sci. U.S.A.* 102 1448–1453. 10.1073/pnas.0409534102 15671180 PMC547876

[B73] Schumacher S. M. Koch W. J. (2017). Noncanonical roles of G protein-coupled receptor kinases in cardiovascular signaling. *J. Cardiovasc. Pharmacol.* 70 129–141. 10.1097/FJC.0000000000000483 28328744 PMC5591054

[B74] Shammas S. L. Crabtree M. D. Dahal L. Wicky B. I. Clarke J. (2016). Insights into coupled folding and binding mechanisms from kinetic studies. *J. Biol. Chem.* 291 6689–6695. 10.1074/jbc.R115.692715 26851275 PMC4807256

[B75] Shukla A. K. Manglik A. Kruse A. C. Xiao K. Reis R. I. Tseng W. C. (2013). Structure of active beta-arrestin-1 bound to a G-protein-coupled receptor phosphopeptide. *Nature* 497 137–141. 10.1038/nature12120 23604254 PMC3654799

[B76] Staus D. P. Hu H. Robertson M. J. Kleinhenz A. L. W. Wingler L. M. Capel W. D. (2020). Structure of the M2 muscarinic receptor-beta-arrestin complex in a lipid nanodisc. *Nature* 579 297–302. 10.1038/s41586-020-1954-0 31945772 PMC7367492

[B77] Sun W. Y. Wu J. J. Peng W. T. Sun J. C. Wei W. (2018). The role of G protein-coupled receptor kinases in the pathology of malignant tumors. *Acta Pharmacol. Sin.* 39 1699–1705. 10.1038/s41401-018-0049-z 29921886 PMC6289378

[B78] Tesmer V. M. Kawano T. Shankaranarayanan A. Kozasa T. Tesmer J. J. (2005). Snapshot of activated G proteins at the membrane: the Galphaq-GRK2-Gbetagamma complex. *Science* 310 1686–1690. 10.1126/science.1118890 16339447

[B79] Thomsen A. R. B. Plouffe B. Cahill T. J. III Shukla A. K. Tarrasch J. T. Dosey A. M. (2016). GPCR-G protein-beta-arrestin super-complex mediates sustained G protein signaling. *Cell* 166 907–919. 10.1016/j.cell.2016.07.004 27499021 PMC5418658

[B80] Thul P. J. Akesson L. Wiking M. Mahdessian D. Geladaki A. Ait Blal H. (2017). A subcellular map of the human proteome. *Science* 356:eaal3321. 10.1126/science.aal3321 28495876

[B81] Tian X. Irannejad R. Bowman S. L. Du Y. Puthenveedu M. A. von Zastrow M. (2016). The alpha-arrestin ARRDC3 regulates the endosomal residence time and intracellular signaling of the beta2-adrenergic receptor. *J. Biol. Chem.* 291 14510–14525. 10.1074/jbc.M116.716589 27226565 PMC4938174

[B82] Tran T. M. Friedman J. Qunaibi E. Baameur F. Moore R. H. Clark R. B. (2004). Characterization of agonist stimulation of cAMP-dependent protein kinase and G protein-coupled receptor kinase phosphorylation of the beta2-adrenergic receptor using phosphoserine-specific antibodies. *Mol. Pharmacol.* 65 196–206. 10.1124/mol.65.1.196 14722251

[B83] Uhlen M. Fagerberg L. Hallstrom B. M. Lindskog C. Oksvold P. Mardinoglu A. (2015). Proteomics. tissue-based map of the human proteome. *Science* 347:1260419. 10.1126/science.1260419 25613900

[B84] van der Lee R. Buljan M. Lang B. Weatheritt R. J. Daughdrill G. W. Dunker A. K. (2014). Classification of intrinsically disordered regions and proteins. *Chem. Rev.* 114 6589–6631. 10.1021/cr400525m 24773235 PMC4095912

[B85] Venkatakrishnan A. J. Flock T. Prado D. E. Oates M. E. Gough J. Madan Babu M. (2014). Structured and disordered facets of the GPCR fold. *Curr. Opin. Struct. Biol.* 27 129–137. 10.1016/j.sbi.2014.08.002 25198166

[B86] Winstel R. Freund S. Krasel C. Hoppe E. Lohse M. J. (1996). Protein kinase cross-talk: membrane targeting of the beta-adrenergic receptor kinase by protein kinase C. *Proc. Natl. Acad. Sci. U.S.A.* 93 2105–2109. 10.1073/pnas.93.5.2105 8700892 PMC39917

[B87] Wright P. E. Dyson H. J. (2015). Intrinsically disordered proteins in cellular signalling and regulation. *Nat. Rev. Mol. Cell Biol.* 16 18–29. 10.1038/nrm3920 25531225 PMC4405151

[B88] Wu C. C. Tsai F. M. Shyu R. Y. Tsai Y. M. Wang C. H. Jiang S. Y. (2011). G protein-coupled receptor kinase 5 mediates Tazarotene-induced gene 1-induced growth suppression of human colon cancer cells. *BMC Cancer* 11:175. 10.1186/1471-2407-11-175 21575264 PMC3112162

[B89] Xiao K. McClatchy D. B. Shukla A. K. Zhao Y. Chen M. Shenoy S. K. (2007). Functional specialization of beta-arrestin interactions revealed by proteomic analysis. *Proc. Natl. Acad. Sci. U.S.A.* 104 12011–12016. 10.1073/pnas.0704849104 17620599 PMC1913545

[B90] Yang F. Yu X. Liu C. Qu C. X. Gong Z. Liu H. D. (2015). Phospho-selective mechanisms of arrestin conformations and functions revealed by unnatural amino acid incorporation and (19)F-NMR. *Nat. Commun.* 6:8202. 10.1038/ncomms9202 26347956 PMC4569848

[B91] Yu S. Sun L. Jiao Y. Lee L. T. O. (2018). The role of G protein-coupled receptor kinases in cancer. *Int. J. Biol. Sci.* 14 189–203. 10.7150/ijbs.22896 29483837 PMC5821040

[B92] Zhu W. Tilley D. G. Myers V. D. Coleman R. C. Feldman A. M. (2013). Arginine vasopressin enhances cell survival via a G protein-coupled receptor kinase 2/beta-arrestin1/extracellular-regulated kinase 1/2-dependent pathway in H9c2 cells. *Mol. Pharmacol.* 84 227–235. 10.1124/mol.113.086322 23690069 PMC3716325

